# A physiologic approach to cord clamping: Clinical issues

**DOI:** 10.1186/s40748-015-0022-5

**Published:** 2015-09-08

**Authors:** Susan Niermeyer

**Affiliations:** Section of Neonatology, University of Colorado School of Medicine, 13121 E. 17th Avenue, Mail Stop 8402, Aurora, CO 80045 USA

**Keywords:** Umbilical cord, Placental transfusion, Resuscitation, Respiration, Infant, Newborn, Infant, Premature

## Abstract

**Background:**

Recent experimental physiology data and a large, population-based observational study have changed umbilical cord clamping from a strictly time-based construct to a more complex equilibrium involving circulatory changes and the onset of respirations in the newly born infant. However, available evidence is not yet sufficient to optimize the management of umbilical cord clamping.

**Findings:**

Current guidelines vary in their recommendations and lack advice for clinicians who face practical dilemmas in the delivery room. This review examines the evidence around physiological outcomes of delayed cord clamping and cord milking vs. immediate cord clamping. Gaps in the existing evidence are highlighted, including the optimal time to clamp the cord and the interventions that should be performed before clamping in infants who fail to establish spontaneous respirations or are severely asphyxiated, as well as those who breathe spontaneously.

**Conclusion:**

Behavioral and technological changes informed by further research are needed to promote adoption and safe practice of physiologic cord clamping.

## Background

*“In the time of Hippocrates the cord was not cut until the placenta was delivered….Since the time of Levret it has been established as a general rule, among accoucheurs, to separate the child from the mother as soon as it has passed through the vulva, and that it is never necessary to wait for the expulsion of the foetal appendages. At first view the conduct of the ancients appears to be more rational and more physiological than that of the moderns; it seems that the placenta ought immediately to follow the foetus, or at least be separated from the uterus before the cord can be prudently cut; that before it is divided, the circulation ought to be permitted gradually to take on its new type, which soon becomes similar to that of the adult; but in reality it is not perceived that the present mode of practice produces the least inconvenience to the foetus, and is certainly better for the mother.”*^1^ – Prof. A.A. Velpeau, 1829 [1]

**Table 1:** Targets for further research on physiologic umbilical cord clamping

Population		
Extremely low birth weight/extremely preterm infants		
Infants with evidence of asphyxia – antepartum/intrapartum		
Infants born in low-resource settings		
Intervention (delayed cord clamping with multiple covariates)		
Antenatal corticosteroid administration before preterm birth		
Type of maternal anesthesia		
Uterine activity (contractions or operative delivery without labor)		
Administration of uterotonic relative to cord clamping		
Onset of respirations relative to cord clamping		
	Spontaneous	
	Assisted ventilation	
Position of infant relative to placenta		
Duration of delay before clamping		
Comparison		
Delay in cord clamping with and without resuscitation		
	Initial steps (drying, clearing airway, specific stimulation to breathe)	
	Positive-pressure ventilation	
		Sustained inflation
		CPAP
		Intermittent positive-pressure ventilation
Umbilical cord milking vs. delayed cord clamping		
	Active milking (length of cord segment, rate, number of passes)	
	Draining of cord segment	
Outcome		
Need for resuscitation		
Physiologic characteristics during postnatal stabilization		
	Temperature	
	Blood pressure	
	Blood glucose	
	Need for volume expanders/pressors (per defined criteria)	
	Regional blood flow – e.g. cerebral	
Hemoglobin/hematocrit/iron status		
Blood volume		
Need for transfusion (per defined criteria)		
Complications of prematurity		
	Intracranial hemorrhage/periventricular leukomalacia	
	Necrotizing enterocolitis	
	Bronchopulmonary dysplasia/duration of supplemental oxygen	
	Patent ductus arteriosus	
Hyperbilirubinemia		
	Premature and term infants	
	Populations at high risk (genetic variations)	
	Settings with limited access to phototherapy	
Polycythemia		
	Term infants and growth-restricted infants	
	Technique-specific differences (delayed clamping vs. umbilical cord milking)	
Neurodevelopment		
	Toddler, preschool, elementary school outcomes	
	Sex-specific differences	
	Correlation with iron status	
	Brain microstructure/development (advanced imaging i.e. MRI)	
Behavior		
	Prevalence/duration of exclusive breastfeeding	
Mortality		
Maternal obstetrical outcomes		
	Physiologic characteristics postpartum	
	Postpartum hemorrhage	
	Intraoperative complications

For centuries, lively debate has surrounded the question of when to clamp and cut the umbilical cord of the newly born infant, and practices have ranged from one extreme to the other. From the time of the Ancient Greeks, midwives have described the value of waiting to clamp the cord until pulsations stop or until the placenta is delivered [1]. This approach is taken to its furthest modern extent in Lotus birth, when the umbilical cord and placenta remain attached to the infant until natural separation at the umbilicus occurs after several days. As Prof. Velpeau pointed out in his Treatise on Midwifery in 1829, a different practice arose among *accoucheurs*, male midwives or obstetricians, who perceived that immediate cord clamping and cutting offered benefit to the mother and posed no “inconvenience” to the newborn [1]. Recently, the obstetrical practice of immediate cord clamping has been modified by policy statements from the American College of Obstetricians and Gynecologists (ACOG), the Royal College of Obstetricians and Gynaecologists, and the The Royal College of Midwives [2–4]. The ACOG statement received endorsement from the American Academy of Pediatrics [5]; the International Liaison Committee on Resuscitation recommended delayed cord clamping for infants who do not require immediate resuscitation [6]; and the World Health Organization (WHO) reiterated their recommendation to delay cord clamping for 1–3 min while initiating simultaneous essential newborn care [7]. Still, all current practice guidelines vary slightly in their emphasis and details, and all suggest that delayed cord clamping may not be feasible or desirable in every situation, especially when immediate resuscitation is required. This review will relate recent experimental physiology data to clinical studies, examine the practical dilemmas faced by clinicians, and identify gaps in knowledge as well as directions for further research to more fully define a physiologic approach to cord clamping.

## Findings

### Physiology of umbilical cord clamping

Physiologic data from experimental animals have changed the frame of reference for umbilical cord clamping from a strictly time-based construct to a more complex equilibrium between the circulatory changes accompanying the onset of respirations and completion of the circulatory and respiratory functions of the placenta. In an accompanying article, Hooper et al. describe in detail the differences in heart rate, right ventricular output, carotid artery pressure and flow, and pulmonary and ductus arteriosus blood flow encountered with immediate cord clamping before onset of respirations vs. delay in cord clamping until after ventilation in anesthetized preterm lambs [8, 9].

**Fig. 1:**
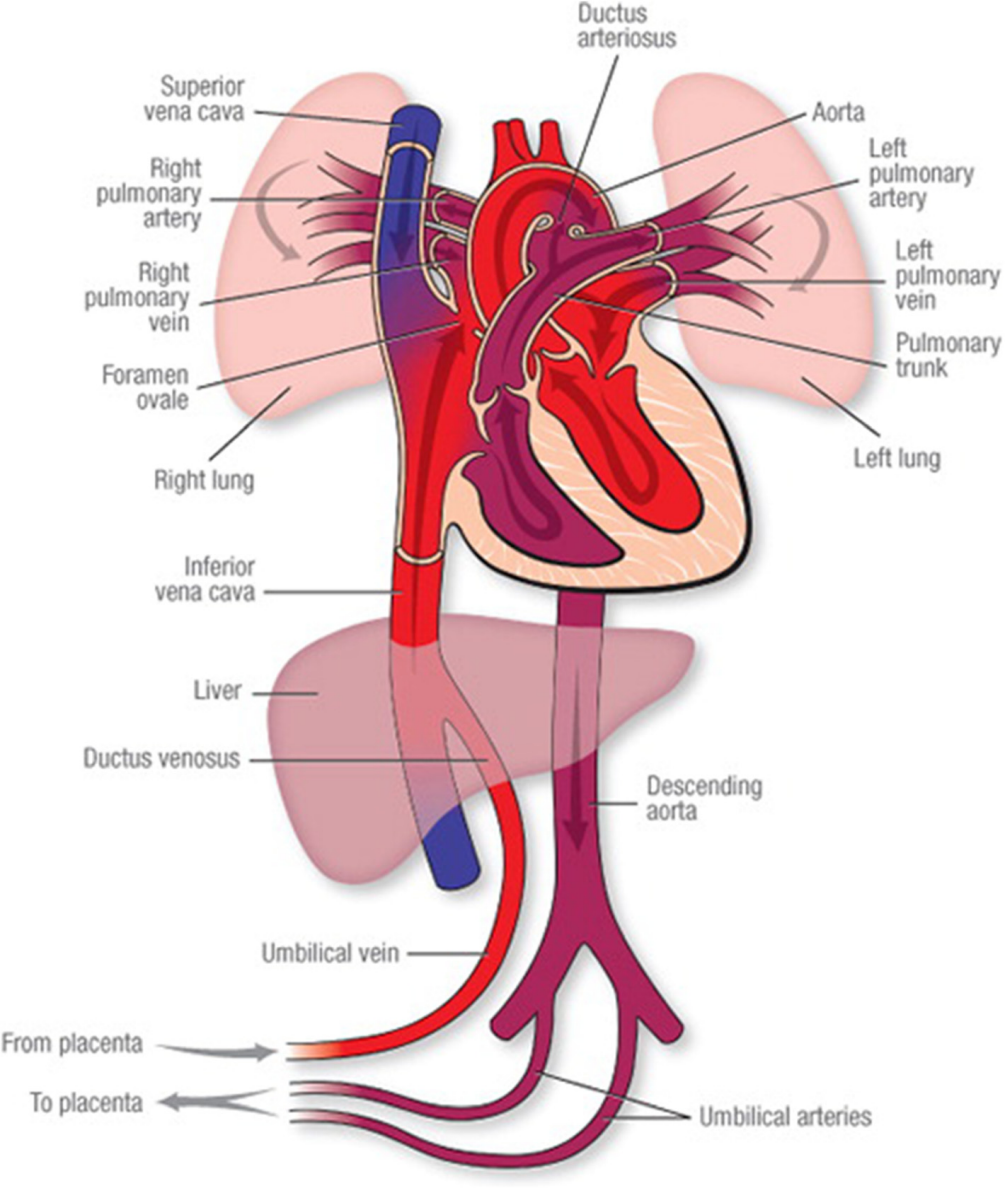
Schematic of the fetal circulation (http://www.heart.org/HEARTORG/Conditions/CongenitalHeartDefects/SymptomsDiagnosisofCongenitalHeartDefects/Fetal-Circulation_UCM_315674_Article.jsp)

At birth, the function of respiration shifts from the placenta to the infant’s lungs, as they expand first with air and then with a large increase in pulmonary blood flow. During fetal life, placental blood passes through the umbilical vein and ductus venosus to the right atrium, where it primarily streams across the foramen ovale to provide preload for the left ventricle (Fig. 1). Systemic fetal venous return also enters the right atrium and passes into the right ventricle; however, only a small percentage of total right ventricular output passes through the lungs. Most right ventricular output diverts via the ductus arteriosus to the descending aorta where it perfuses fetal organs or returns to the placenta via the umbilical arteries. When breathing begins, much more of the right ventricular output flows to the lungs and placental blood maintains ventricular preload. As the pulmonary circuit fills, pulmonary blood return to the left atrium gradually increases to serve as preload for the systemic circulation. In the process, pulmonary vascular resistance falls, right heart pressure falls, and the foramen ovale functionally closes [10, 11]. When the umbilical cord remains intact as a healthy infant begins breathing, the shift in respiratory function from placenta to lungs is accompanied by a physical shift in blood volume from the placenta to the newly born infant to maintain circulatory equilibrium as the pulmonary vascular bed opens.

If the umbilical cord is clamped before breathing begins, systemic blood pressure rises with loss of the low-resistance placental circuit and still-high pulmonary vascular resistance preventing left-to-right flow through the ductus arteriosus. There follows a loss of preload to the left ventricle which results in a steep fall in cardiac output [12]. If there is delay in establishing respirations, hypoxemia also ensues, potentially resulting in postnatal hypoxic-ischemic conditions. Compensatory mechanisms move circulating volume from the peripheral to central circulation. Only when pulmonary vascular resistance falls does pulmonary blood flow increase through right ventricular outflow and left-to-right shunting through the ductus arteriosus. Pulmonary venous return to the left atrium then restores left ventricular preload, cardiac output, and systemic pressure [9, 13].

**Fig. 2:**
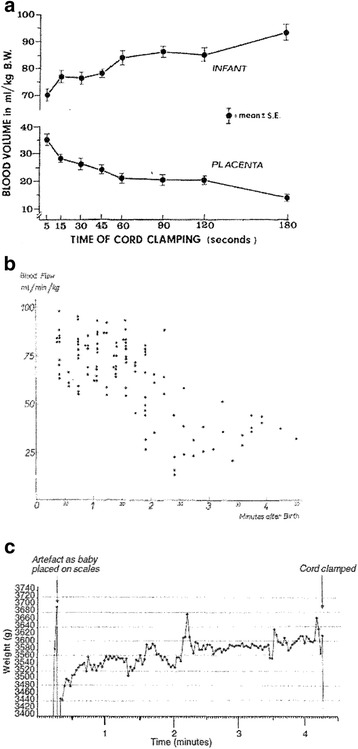
Various lines of evidence (**a** residual placental blood volume, **b** umbilical blood flow by dye dilution, **c** infant weight) demonstrating continuation of umbilical blood flow for several minutes after birth (permission requested) [57, 18, 17]

Reaching the point of circulatory equilibrium in the transition from placental to pulmonary respiration requires a variable amount of time, depending on the individual circumstances at birth. Various lines of data from early physiological studies suggest that the transition usually lasts several minutes [14]. In ventilated lambs, pulmonary blood flow reaches a maximum only after 5–10 min [15]. Measurement of residual placental blood volume in human infants describes rapid transfer of blood initially, followed by slower rate of transfer documented through 3 min or more [16] (Fig. 2). Measurements of actual umbilical vein flow by dye dilution follow a similar pattern in healthy term infants, with high flow in the first 2 min, followed by a variable decrease [17]. More recent studies show a similar pattern of increase in infant weight after birth when the umbilical circulation remains intact [18]. Umbilical Doppler blood flow patterns immediately after birth are highly variable, but flow may continue up to 10 min [19]. Electrical impedance measurements show an increase in cardiac output through 3–5 min in the majority of infants [20]. Experimental and clinical physiological data are generally consistent in suggesting that reaching circulatory equilibrium requires several minutes, even under ideal circumstances.

A single large, population-based observational study of infants in a resource-limited setting in Tanzania reported an association between the timing of umbilical cord clamping relative to the onset of spontaneous respirations and the outcomes of death or admission to a special care area [21]. The cohort of 12,730 term and preterm infants was selected on the criterion that all established spontaneous respirations and received only routine care. They were judged to be healthy and strong by the delivering midwives. Independent observers documented the timing of key interventions and infant responses at all deliveries. Variation in the time of umbilical cord clamping occurred as the facility transitioned from the previous practice of immediate cord clamping to a new routine of cord clamping delayed for 1–3 min. Special care in the neonatal area was limited to administration of antibiotics and intravenous fluids. Logistic modelling showed the risk of death/admission to the special care area by 24 h was consistently higher if cord clamping occurred before spontaneous respirations. Risk of death or admission decreased by 20 % for every 10-s delay in cord clamping after spontaneous respiration up to 2 min. Death/admission occurred more frequently in infants of low birth weight (<2500 g) and preterm gestational age; however the described relationship held in both normal weight and low birth weight infants. In a setting where cardiorespiratory or even general supportive care was not available to buffer physiologic derangements, the impact of cord clamping before onset of respirations manifested as increased risk of death and instability, and delay in cord clamping through 2 min had a dose-dependent effect of decreasing that risk.

Although the physiological data and clinical data from resource-limited settings indicate a clear and important relationship between onset of respirations and the timing of cord clamping, the situations faced by clinicians are complex and variable. Available literature does not yet provide sufficient high-quality evidence to address management with complications such as intrapartum asphyxia, fetal growth restriction, or extreme prematurity. While some data on physiologic outcomes exist, primary outcomes have more commonly related to hemoglobin/hematocrit and volume of placental transfusion. Thus, there are gaps in the existing evidence to guide management, and clinicians face a number of dilemmas, including:

- Timing – What is the optimal time to clamp the cord in term and preterm infants who breathe spontaneously, fail to establish spontaneous respirations, or are severely asphyxiated? Should resuscitative interventions be performed during the interval before clamping to stimulate breathing or begin assisted ventilation?
- Technique – does umbilical cord milking offer equivalent or superior outcomes to delayed cord clamping? What is the interplay of umbilical cord milking and onset of respirations?
- To be determined – what gaps in knowledge, skills, and technological support impede the adoption and safe practice of physiologic cord clamping in the clinical setting?

### Timing: optimizing the relationship between cord clamping and onset of respirations

*“If a careful attention be paid to what happens after an ordinary birth, it will be seen that the pulsations grow weaker and soon disappear in the cord, beginning at the placenta, and that after a few minutes it may be cut without being followed by the least hemorrhage. This remarkable phenomenon which is attributed to the change of direction of the iliac arteries, and to the difficulty experienced by the blood in passing into the aorta through the ductus arteriosus, and into the cord through the umbilical arteries, always takes place where everything occurs in a natural and regular order, but in reality depends upon the circumstance that the attractive force exerted by the placenta upon the blood, is replaced by that of the respiratory organ, and that the after-birth is no longer anything more than an inert substance, without vitality, which is abandoned by the blood, as it abandons a gangrenous or asphyxiated limb.” *2

The clinician is faced with a number of uncertainties before the delivery of a newborn. Will the lungs be mature? Will the baby cry and breathe spontaneously? Will an acute intrapartum hypoxic-ischemic event or an obstetrical circumstance preclude delayed cord clamping? The answers to each of these may become apparent only at the moment of delivery, yet they all potentially influence management of umbilical cord clamping and immediate care of the newly born infant. The available evidence relating to physiologic outcomes for term and preterm infants who breathe spontaneously can help guide clinical practice. The limited data from babies who are born in either primary or secondary apnea help to frame gaps in knowledge and suggest practical approaches to management and further research.

#### Spontaneous cry in term infants

The healthy term infant who cries spontaneously and breathes well often accomplishes the crucial first moments of circulatory transition with an intact umbilical cord, even when clamping is not deliberately delayed. Early physiological studies of residual placental blood volume demonstrated that a relatively larger proportion of the fetoplacental blood volume resides in the fetus at term as compared to earlier in gestation. Spontaneous cry before umbilical cord clamping significantly increases the volume of placental transfusion and hematocrit [22]. The curve of blood volume transferred vs. time as constructed by Yao highlights that more than half of the total blood transfer occurs within the first minute [16]. Among healthy term infants delivered vaginally, position at the perineum or on the mother’s abdomen/chest does not significantly affect the volume of placental transfusion when clamping is delayed until 2 min [23]. Umbilical vein flow, as measured by dye dilution, is fastest in the first 1–2 min and slows variably thereafter [17]. Doppler ultrasound of umbilical blood flow patterns immediately after birth reveals highly variable flow duration and direction in both the umbilical artery and vein. Flow continues longer than previously described, with flow documented in some cases at the time of cord clamping between 5 and 10 min. In addition, the presence of cord pulsations does not necessarily signify ongoing flow, and cessation of pulsations can occur with or without flow [19].

Most literature examining clinical outcomes of delayed cord clamping in term infants reports primary outcomes related to volume of placental transfusion and/or hematocrit and relatively few secondary physiological endpoints. Meta-analysis of trials in term infants confirms that not only are acute measures of blood volume and hematocrit generally improved among infants with delayed clamping, but indices of iron status in infancy are also improved [24]. This has important implications in the prevention of iron-deficiency anemia and the associated sequelae of impaired cognitive, motor and behavioral development. While the global burden of iron deficiency anemia is greatest in sub-Saharan Africa and Southeast Asia, improvement in hematologic indices occurs even in highly developed industrialized countries such as Sweden [25]. Although short-term neurodevelopmental outcome showed no difference, follow-up at 4 years of a cohort of term Swedish infants with clamping delayed until 3 min shows improved processing speed, fine motor and personal-social scores compared to infants with immediate clamping [26, 27]. Another behavioural outcome of potential significance is the observation of improved rates of exclusive or predominant breastfeeding after hospital discharge [28]. One postulated linking mechanism is improved physiologic stability and alertness in the hour immediately after birth, promoting successful early initiation of breastfeeding, which in turn increases the duration of breastfeeding and the likelihood of exclusive breastfeeding through 4 months of life [29]. Studies of skin temperature in the days after birth support improved physiologic stability in late-clamped term infants and give indirect evidence for peripheral cutaneous vasoconstriction among those with early clamping. Infants with cord clamping delayed until pulsations stopped (average 3 min and 38 s) show significantly higher heel and palm temperatures than the group with immediate clamping, but no difference in epigastric or rectal temperatures [30]. Physiologic studies of cardiac and haematological indices show transient alterations consistent with increased circulating red cell volume but no systematic evidence of increased need for special care (i.e. for hyperviscosity/polycythemia or respiratory distress) [31–34].

**Fig. 3:**
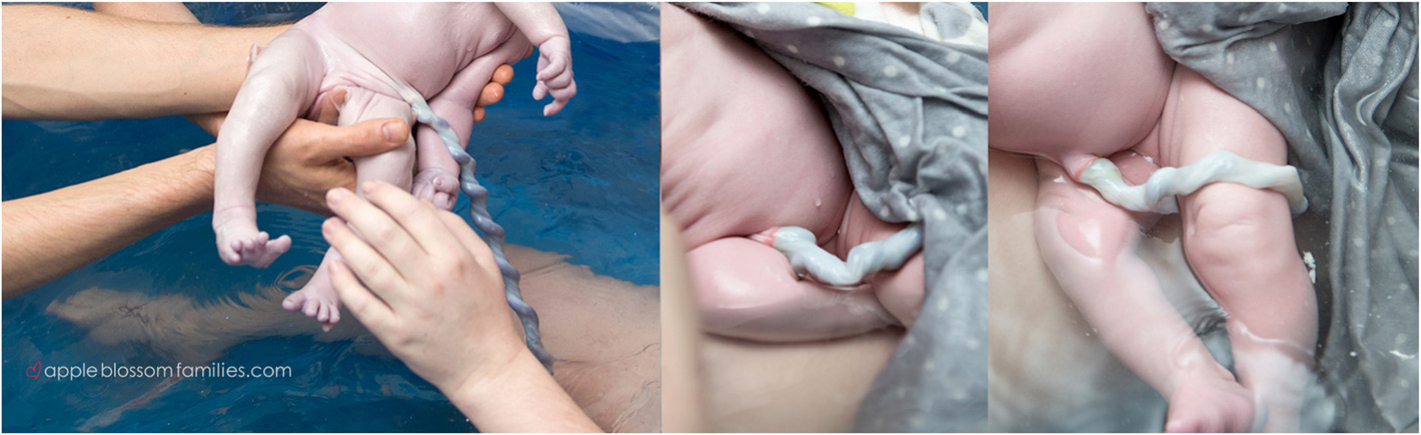
Change in appearance of the umbilical cord from birth, to 12 and 23 min after birth, with umbilical cord intact and completion of third stage of labor at 30 min (appleblossomfamilies.com, Morag Hastings, photographer)

In practice there is evidence to support a delay of two minutes or more, while providing routine care, before clamping the umbilical cord of the healthy term newborn. There is presently no accepted clinical sign indicating that circulatory equilibrium has been achieved between the newly born infant and the placenta. The midwifery literature describes “flattening” of the cord (Fig. 3) as a correlate for completion of the placental transfusion [35] and cessation of pulsation also has been used as an endpoint. Position at the introitus or on the mother’s abdomen appears equivalent for the vigorous term infant [23]. Routine care at vaginal delivery, including thorough drying, any necessary clearing of the airway, thermal protection (skin-to-skin contact with mother), and monitoring of breathing, can occur during the delay before clamping and cutting the cord. At caesarean section, the infant can be placed between the mother’s legs on the sterile field and routine care can be provided with warmed, sterile towels for drying. Although skin-to-skin contact cannot occur during the delay at caesarean section, the infant can be positioned skin-to-skin with the mother above the anesthesia barrier drape immediately after dividing the cord. This requires that the mother be alert under regional anesthesia and supported by the continuous presence of a nurse to monitor the infant’s condition and safe positioning [36].

#### V*ariable establishment of respirations in preterm infants*

*“I once received a human foetus, at the sixth month of pregnancy, enclosed within its membranes. The umbilical arteries continued to beat strongly as long as the membranes were unruptured; but they fell into inertia as soon as the lungs and chest, upon coming in contact with the air, attempted to perform some respiratory movements. And do we not every day see the blood flow or stop spontaneously in the same child, accordingly as the respiration is free or embarrassed?”*^3^

The preterm infant who is healthy may cry and breathe spontaneously or may have delayed onset of spontaneous respirations or shallow, irregular breathing after birth [37]. When general anesthesia at preterm delivery is necessary for maternal medical or obstetrical conditions, the newly born infant may be completely apneic, although not asphyxiated, and require positive-pressure ventilation for lung expansion. Preterm infants manifest increased vulnerability to end-organ injury in the cerebral and intestinal circulations (intracranial haemorrhage and necrotizing enterocolitis), making ventilation of the lung prior to umbilical cord clamping and smooth cardiovascular transition theoretically even more desirable. Yet, very few human studies have explored the physiologic changes around respiration and cord clamping in preterm births.

Much of the literature reporting clinical outcomes of delayed cord clamping in preterm infants reports primary outcomes related to volume of placental transfusion and hemoglobin/hematocrit with some additional secondary physiological endpoints. Multiple small, randomized controlled trials and several meta-analyses confirm higher hematocrit at birth and decreased need for transfusion during hospitalization [38]. There is also evidence for improved cardiovascular stability, with higher mean blood pressure at 1 and 4 h after birth [39–42], higher measured blood volume [43, 44] and higher superior vena cava flow [45, 46]. Two major preterm morbidities, intracranial hemorrhage (all grades) and necrotizing enterocolitis, occur less frequently among infants who received delayed cord clamping. However, severe intracranial haemorrhage (grades III and IV) and mortality to discharge do not differ between groups. Temperature on admission to a newborn area does not differ; the rate of hyperbilirubinemia treated with phototherapy and the peak serum bilirubin are higher after delayed cord clamping, but criteria for treatment vary widely [38]. Two randomized trials have reported neurodevelopmental outcome (Bayley II scores), at 7 or 18–24 months age, showing no difference in Mental Developmental Index < 70, but very wide confidence intervals [47].

Two large observational studies with comparison groups report intermediate physiologic outcomes as part of quality improvement programs introducing delayed cord clamping for preterm infants [48, 49]. Very-low-birth-weight (VLBW) infants with delayed cord clamping received less delivery room resuscitation (supplemental oxygen, bag and mask ventilation, intubation, compressions, medications) compared to the immediate cord clamping group (61 vs. 79 %, *p* = 0.01), but there was no difference between groups among low-birth-weight (LBW) infants (30 vs. 27 %). The second study reported no difference in the need for ventilation in the delivery room among infant 24–34 weeks gestation [49]. Blood pressure in the first 2 days of life was higher in LBW late-clamped infants, but not different between the VLBW groups [48]. Mean temperature was in the normal range but higher for infants compliant with delayed cord clamping, and the incidence of temperature < 36.3C was decreased in this group [49]. In both studies, health care providers could clamp the cord if they felt it necessary; 6 of 249 infants [48] had delayed clamping abandoned to begin immediate resuscitation, and 12 of 236 infants [49] remained apneic and inactive after 20 s of assessment, prompting delayed clamping to be abandoned.

**Fig. 4:**
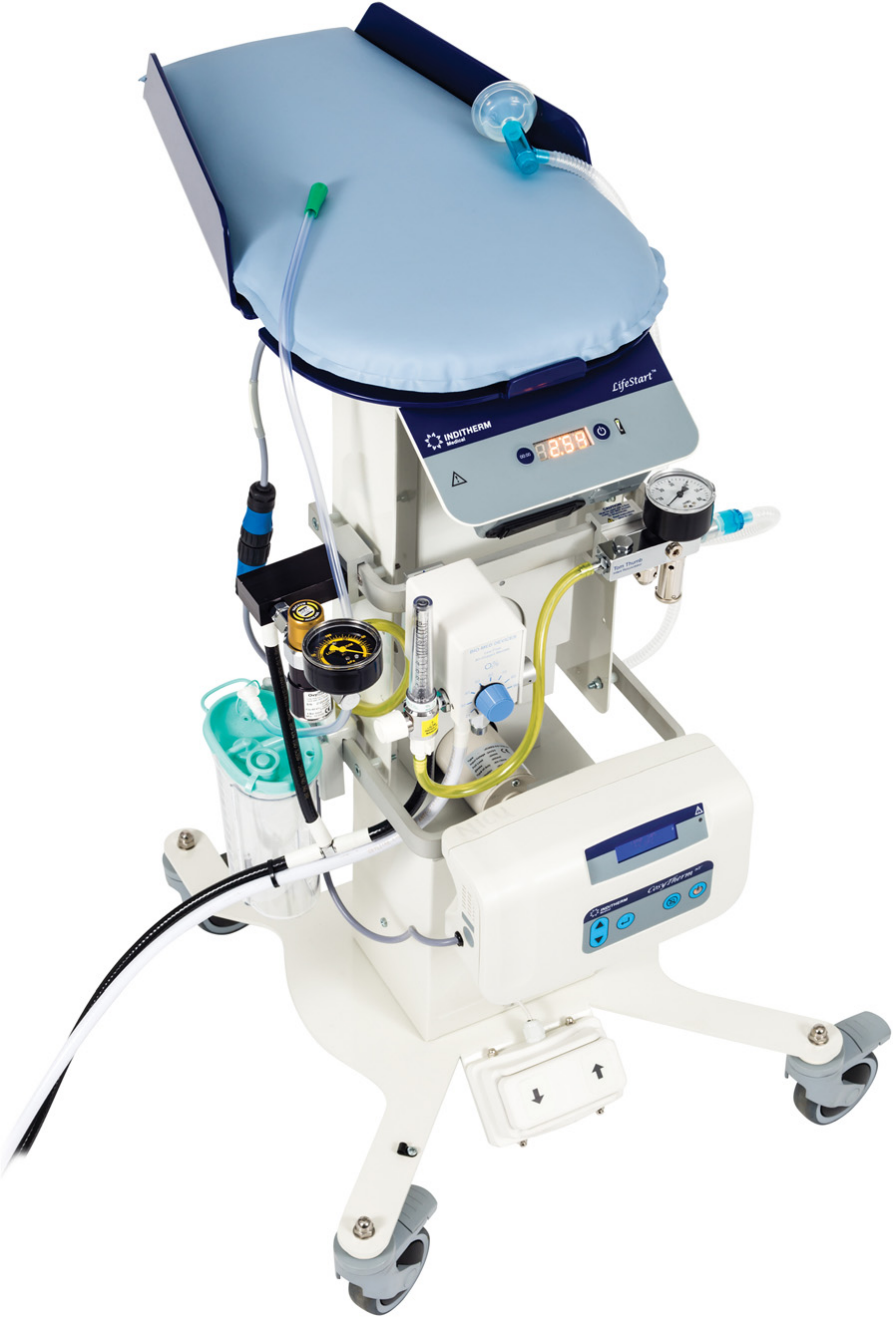
Platform for resuscitation at the beside with umbilical cord intact (LifeStart, Inditherm plc, Rotherham, United Kingdom)

For the clinician, there is insufficient strong evidence to guide either the length of the delay before clamping the umbilical cord or the extent of resuscitative interventions provided to preterm infants during a delay. In many trials, no resuscitative intervention was provided; in other studies, infants were provided the initial steps of resuscitation (drying, positioning and clearing the airway as needed, specific stimulation to breathe i.e. rubbing the back) [43, 48]. Theoretical and practical challenges merit carefully controlled trials of beginning positive-pressure ventilation with intact umbilical circulation. Although positive-pressure ventilation may be necessary to aerate the lung and increase pulmonary blood flow, experimental animal studies suggest excessive end-expiratory pressure or mean airway pressure may not only cause lung injury, but may also impair cardiovascular transition and result in potential cerebral injury [50]. The physical arrangement necessary to provide positive-pressure ventilation calls for collaboration between providers caring for the mother and those caring for the newly born infant. A small trial has demonstrated the feasibility and acceptability of a compact, adjustable resuscitation platform (Fig. 4) which can be positioned close to the perineum at normal and assisted vaginal birth or the abdominal incision at caesarean section [51]. Alternatively, a flat, dry, firm surface can be positioned at the perineum on the unbroken delivery bed or on the mother’s legs at caesarean section. Use of a t-piece device for ventilation or CPAP limits the equipment introduced into the field of the delivery or the sterile field at caesarean section. Delayed cord clamping may require modification of routines for thermal protection with polyethylene wrap or bags. However, contemporary observational cohorts suggest that improved placental transfusion may help maintain body temperature [48, 49]. Randomized controlled trials using a package of interventions addressing cord clamping, initial support of breathing/positive-pressure ventilation, low supplemental oxygen, and thermal protection will be challenging, but vital to ascertain the real potential of these interventions combined.

#### Asphyxia and secondary apnea in term or preterm infants

*“Where there is reason to believe that the placenta still maintains a part of its natural relations with the womb, and especially where there is still some tremour, some pulsation in the cord, we may follow the advice…not to cut it too soon; but if the womb be well contracted, if the adhesions of the placenta be evidently destroyed, it would be better to separate the fcetus at once from its mother.”*^4^

The asphyxiated term or preterm infant presents unique physiological circumstances around umbilical cord clamping. Early literature suggests that antepartum asphyxia is accompanied by in utero transfer of blood volume from the placenta to the fetal circulation [52–54]. Data from dye-dilution studies of the umbilical circulation in term asphyxiated infants support lower flow rates immediately after birth compared to healthy controls [55]. In contrast, acute intrapartum asphyxial events, such as tight nuchal cord or shoulder dystocia, may be accompanied by cord occlusion and hypovolemia. In either setting, with severe hypoxia-ischemia, the myocardium and the cerebral circulation may be particularly vulnerable to further insult from immediate clamping of the cord. The resultant loss of preload and transient decrease in cardiac output are likely to produce bradycardia, swings in cerebral perfusion, and further interruption in blood flow to the renal, gastrointestinal, and pulmonary circulation. Although the volume of blood transferred after birth may be reduced or increased compared to non-asphyxiated infants, the maintenance of a patent umbilical circulation at least until ventilation has been established may limit further injury. However, chest compressions in the setting of intact umbilical circulation theoretically could fail to generate the diastolic pressure vital for coronary perfusion and return of spontaneous circulation because of the large, low-resistance vascular bed in the placenta [56]. There is also theoretical concern that hydrostatic pressure resulting from the relative position of infant and placenta may exert a substantial effect on the direction/volume of blood transfer in the setting of asphyxia [57].

Management of umbilical cord clamping in the asphyxiated term or preterm infant has received little attention in recent randomized controlled trials; usually the need for immediate resuscitation has constituted a criterion for exclusion. The logistic challenges of conducting an extensive resuscitation, including possible need for immediate intubation, chest compressions, and umbilical line placement for medication administration, argue for use of a specialized resuscitation platform for initial management proximate to the field of delivery. Once the initial steps of resuscitation and lung expansion have been accomplished, the infant can be relocated for more extensive interventions.

### Technique: cord milking as an alternative to delayed cord clamping

*“In the time of Aristotle the midwives were in the habit of forcing the blood contained in the cord into the belly of the fcetus before they tied it, and pretended by means of this practice, which has been revived at the commencement of the present century, to restore strength and vigour to feeble children.”*^5^

Cord milking offers an alternative to delayed cord clamping for facilitation of placental transfusion. Cord milking typically involves compressing the blood from a 20–30 cm segment of cord into the infant over 2–3 s; usually this procedure is repeated three times, allowing the umbilical vessels to refill from the placental side between milking [58]. To maximize transfer of blood, coils in the umbilical cord must be untwisted first. When there is great urgency, the cord can be immediately clamped close to the placental side, suspended over the infant on the resuscitation platform, and milked or passively drained as resuscitative interventions begin. Measurement of residual blood volume in the umbilical cords of extremely preterm infants estimates the volume contained in a 30-cm segment of cord at approximately 17 mL/kg [59]. The residual volume tended to be reduced in the cords of infants with low weight for gestational age.

The evidence base for umbilical cord milking is more limited than that for delayed umbilical cord clamping. Meta-analysis of five small randomized controlled trials in preterm infants < 33 weeks shows higher initial levels of hemoglobin and hematocrit, as well as lower incidence of intracranial haemorrhage of all grades in infants who received umbilical cord milking as compared to immediate clamping [60]. One recent trial reports that preterm infants in the umbilical cord milking group had higher heart rate over the first 2 min, higher oxygen saturation in the first minutes with less supplemental oxygen, and reduced need for supplemental oxygen at 36 weeks [61]. Preterm infants < 32 weeks born by caesarean delivery show higher superior vena cava flow in the first 12 h with umbilical cord milking as compared to 45–60 s of delay in cord clamping [62]. The meta-analysis finds no difference in mortality before discharge, hypotension requiring volume or inotropes, need for transfusion, severe intraventricular haemorrhage, peak serum bilirubin or need for phototherapy [60]. Only one study followed infant to 24 months for developmental outcome, finding no difference in rates of disability [47]. Two small randomized controlled trials in infants > 35 weeks report higher hemoglobin immediately after birth. Of 224 total infants, none had symptomatic polycythemia. There was no difference in the peak serum bilirubin or the need for phototherapy [63, 64].

In practice, umbilical cord milking offers the primary advantage of speed. The actions can be completed in a matter of seconds, the maneuvers are readily accomplished at caesarean section, and the baby can be immediately transferred to the pediatric team for any needed resuscitation. Entire surgical teams do not need to pause during a caesarean section, and the obstetrical provider at a vaginal delivery can immediately move to controlled cord traction. The action of milking the cord requires some standardization of practice, however, as the helical spirals of the cord create obstruction to blood flow if not untwisted prior to milking. The length of the segment milked, speed, and number of repetitions of milking have been established by pragmatic choice rather than controlled experiments. Transfer of blood occurs as a bolus rather than a process of equilibration as with delayed cord clamping; milking often is accomplished in 15 s or less [61]. If respirations have not been established prior to clamping the cord, the volume of blood retained in the infant’s circulation may not be as large as if the pulmonary circulation has expanded. Hemodynamic fluctuations similar to those described with immediate cord clamping theoretically could occur; the hemodynamic significance of retrograde flow in the umbilical arteries also is unclear. Additional concerns arise around potential volume overload in infants who have experienced hypoxic-ischemic events during labor and increased products of endothelial activation entering the infant’s circulation with milking of the umbilical arteries [65].

### To be determined: gaps in knowledge and practice

*“Instead of coming into the world pale, anemic, or exsanguinous, the child is sometimes born in quite a contrary condition; its skin is of a bluish red or liver colour, of various degrees of intensity, especially on the face, and appears as if thickened…..The apoplectic state is met with, especially in strong children, after long and difficult labours, the application of the forceps, and pelvis labours, either spontaneous or artificial; where the child has remained for several hours under the influence of the uterine contractions after the discharge of the waters; where it has presented in a bad position; where it is too large to pass with ease through the various passages; where a loop of the cord strictures its neck, or is itself in any way compressed, and particularly where any of these accidents occur coincidently with a previous plethoric state. When a child is born in this state we should make haste to disengorge its vascular system…” *6

#### When is physiological cord clamping not feasible or beneficial?

Widely varying conditions have been considered as exclusions from delayed cord clamping or umbilical cord milking in published trials. Hemorrhagic complications during labor (i.e. placental abruption and placenta previa) and cord abnormalities or accidents (true knot, prolapse, avulsion) may physically interrupt placental transfer. Tight nuchal cord which cannot be reduced conventionally may be reduced by using the somersault maneuver, permitting delayed clamping or cord milking [66]. In multiple gestations with evidence of placental connections (monochorionic placentation, twin-twin-transfusion syndrome) or discordant growth, facilitating placental transfusion poses theoretical risk to the second twin and potential to exaggerate polycythemia/hypervolemia. Hemolytic disease has been viewed as a contraindication because delayed clamping increases the volume of sensitized red blood cells; however the relative risks/benefits of hypovolemia and hemodynamic lability at birth vs. larger bilirubin load have not been carefully studied.

Questions arise around a number of maternal and fetal conditions which might represent relative contraindications to facilitated placental transfusion. Growth restriction, especially if a result of chronic intrauterine hypoxia, may be associated with higher risk for polycythemia and hyperviscosity. Maternal diabetes and pre-eclampsia also represent circumstances associated with intrauterine hypoxia. Non-vigorous infants born through meconium-stained amniotic fluid have historically undergone immediate intubation for tracheal suctioning, prompting immediate cord clamping. Programs focusing on prevention of maternal-to-child transmission of HIV previously advocated immediate clamping as part of the package of interventions; in the absence of any evidence or logical mechanism of increased viral transmission, WHO recommendations now explicitly advocate delayed cord clamping in this situation [7].

#### What factors determine the volume/rate of blood transfer and what clinical indicators can aid in establishing the point of hemodynamic equilibrium?

During either delayed cord clamping or umbilical cord milking in the clinical setting there is little certainty about the volume, rate, or direction of blood transfer. The onset of spontaneous respirations and opening of the pulmonary vascular bed are now recognized as major determinants of placental blood transfer. However, other factors have been demonstrated to influence the equilibrium of placental transfusion, including uterine contractions (or their absence at caesarean section), administration of uterotonics, physical position of the newly born infant relative to the placenta, gestational age, and antepartum/intrapartum hypoxic-ischemic conditions [67]. A variety of experimental techniques have been used in human infants to document blood flow and net transfer. Highly accurate scales have been used to document the time course and estimate the volume of placental transfusion with delayed cord clamping [18]. In the 1960s impedance plethysmography was used to study the relative contributions of lung aeration and pulmonary blood flow during delayed cord clamping [68]. Modern electrical impedance tomography (EIT) converts impedance signals into images and may offer the prospect of studying lung recruitment and the dynamics of delayed cord clamping simultaneously and noninvasively. Doppler flow measurement has proven feasible for the measurement of umbilical cord flow, as well as cardiac output, regional blood flow, and tissue perfusion. Near-infrared spectroscopy offers insight into cerebral hemodynamics with non-invasive, bedside methods [11, 69]. Experimental animal studies offer the possibility of multiple simultaneous and direct measures of blood flow, pressure, volume as well as advanced imaging using such techniques as scintigraphy and magnetic resonance imaging (MRI). More attention could also focus on description of clinical correlates of adequate placental transfusion, such as capillary refill in the infant or appearance of the umbilical cord. In parallel with lung recruitment, the timing of cord clamping as a patient-defined strategy deserves further research to define useful measures and clinical signs of physiological equilibrium [70].

#### What changes in infrastructure and human behaviour are necessary?

While uncertainties still exist around the best approach to achieve physiologic cord clamping, incremental changes in practice are now possible [71, 49, 48]. The model of gradual transition in circulation and respiratory function from the placenta to the lungs should serve as the model for collaboration between obstetrical and pediatric providers at a birth. Both disciplines need to communicate and formulate a plan before birth to accomplish a smooth transition; both need to be involved in monitoring third stage of labor in the mother and signs of circulatory transition and equilibrium in the newly born infant; both need to promptly detect and respond to deviations from the normal postnatal course. Waiting is difficult, especially waiting without action for a process that is largely invisible and incompletely understood. Active monitoring of umbilical cord appearance/pulsatility as well as uterine contractions/placental detachment and active provision of routine care or the initial steps of resuscitation turn passive waiting into action and provide shared learning and experience with clinical signs of physiologic transition and equilibrium. Although umbilical cord milking also eliminates passive waiting, controlled comparisons of delayed cord clamping vs. umbilical cord milking are necessary before it can be established that cord milking and rapid clamping/cutting pose “not….the least inconvenience” for the newly born infant.

Delay in term infants has often been limited to one minute, which may be insufficient to complete the process of placental transfer to a point of equilibrium. Observational studies in term infants to determine optimal timing should examine carrying out the steps of routine care with the newly born infant on mother’s abdomen to safely prolong the delay and set the stage for early initiation of breastfeeding. However, in many settings, maintenance of skin-to-skin contact in the first hour and facilitation of breastfeeding demand considerable change in provider workflow to provide adequate support and monitoring of mother and baby.

Delay in umbilical cord clamping of premature infants in many studies has been in the range of 30–45 s only; these studies very likely have not captured the full potential benefit of delayed cord clamping to preterm infants. Assuring thermal protection and providing the initial steps of resuscitation to preterm infants during delay in cord clamping can safely prolong the delay and possibly achieve onset of respirations in the majority of infants. Performing the initial steps of routine care or resuscitation during delay deserves comparison with umbilical cord milking.

Delivery of positive-pressure ventilation to achieve lung expansion before umbilical cord clamping calls for re-design of communication and the physical interaction between obstetric and pediatric providers, as well as the equipment for resuscitation. Although equipment for suctioning and ventilation can be readily adapted to space and sterility constraints, monitoring currently proves more cumbersome. Disposable colorimetric carbon dioxide detectors may offer a useful summative indicator for both ventilation and pulmonary blood flow; delivery of carbon dioxide to the alveoli requires pulmonary blood flow and delivery of carbon dioxide to the detector requires unobstructed pulmonary ventilation. Color change has been shown to be useful in detecting airway obstruction during bag and mask ventilation and predictive of restoration of normal heart rate in the setting of bradycardia immediately after delivery [72]. There is considerable potential for further development of monitoring, such as transthoracic impedance and quantitative end-tidal carbon dioxide, that would give real-time information on progress of the physiologic transition after birth.

In resource-limited settings, the balancing outcomes of temperature stability and hyperbilirubinemia deserve close consideration. Thermal protection during delay in umbilical cord clamping may require different strategies where control of environmental temperature and use of chemical warming mattresses are limited. It is unclear if delayed cord clamping in preterm infants would increase demand for phototherapy beyond available capacity in areas where specialty care is limited.

### Directions for further research

#### What are the broader implications of physiologic umbilical cord clamping for neonatal clinical research?

Research is now needed to investigate how the beneficial effects of physiologic transition can be maximized (Table 1). With respect to umbilical cord clamping, this means re-orientation of research to clearly document the timing of cord clamping in relation to the onset of respirations in all studies and to place new emphasis on collection of immediate and intermediate cardiovascular outcomes as well as long-term neurodevelopmental outcomes in both term and preterm infants. The results of large randomized clinical trials comparing delayed and immediate clamping are needed to improve the low quality of evidence now aggregated from multiple small trials with variable patient populations, few extremely preterm infants, and variable interventions and outcome measures. The Cord Trial in the United Kingdom is comparing clamping within 20 s to clamping after at least 2 min among births < 32 weeks gestation [73]. The Australian Placental Transfusion Study compares clamping within 10 s to clamping after 1 min with the infant held below the level of the placenta. Comparative trials of delayed cord clamping vs. cord milking can help define the relative advantages of each method. Experimental physiology studies of umbilical cord milking could help further define the hemodynamics of that practice. Trials that specifically examine initiation of positive-pressure ventilation and/or continuous positive airway pressure with the umbilical cord intact are underway. Packages of interventions that include other aspects of physiologic transition (thermal control, minimizing oxygen exposure and intubation, facilitating lung recruitment with positive-pressure ventilation, skin-to-skin contact for moderate and late preterm infants) should be tested for their potential to achieve more-than-additive benefit.

## Conclusions

The existing literature on delayed cord clamping and cord milking has consistently demonstrated benefit in facilitating placental transfusion. Improved physiologic stability in transition (blood pressure), reduced need for transfusion, and lower incidence of intracranial haemorrhage and necrotizing enterocolitis are valuable improvements in outcome. Several studies, of both delayed cord clamping and cord milking, have shown reduced need for resuscitative interventions. Although these outcomes fall short of the overarching goals of improved neurodevelopmental outcome and reduced mortality in intensive care settings, they offer promise. And, under circumstances where medical support available to newly born infants is severely limited, delayed cord clamping shows a positive relationship with decreased mortality.

Recent experimental and population-based studies highlight the interaction between onset of respirations and timing of umbilical cord clamping. These have changed the concept of delayed cord clamping from one of a defined time interval only to one of facilitation of physiologic transition after birth. Before clear guidance is available for clinicians on how best to facilitate this transition, more research is needed. For the term infant who breathes spontaneously, a delay of at least 2–3 min appears necessary to optimize circulatory transition and placental transfusion. More evidence is needed about the interventions that should be performed during delay in cord clamping among infants who fail to breathe after birth. Preterm and very low birth weight infants as well as infants who have experienced hypoxic-ischemic events are of special interest, as they have generally been excluded from past studies. Data on umbilical cord milking are limited, and comparison between delayed cord clamping and cord milking is especially important.

## Abbreviations

ACOG: American College of Obstetricians and Gynecologists
CPAP: Continuous positive airway pressure
EIT: Electrical impedance tomography
MRI: Magnetic resonance imaging
WHO: World Health Organization

## Footnotes

1 Velpeau, AA. Traité elementaire de l'art des accouchements; ou, Principes de tokologie et d'embryologie [Elementary treatise on the art of midwifery; or, Principles of tokology and embryology], 3^rd^ Edition English translation of the 1829 French text, Philadelphia: Lindsay & Blakiston, 1845, page 550, section 1209.

2 Ibid, page 552, section 1213.

3 ^c^Ibid, page 553, section 1213.

4 Ibid, page 559, section 1222.

5 Ibid, page 551, section 1212.

6 Ibid, page 562-3, section 1227–9.
